# Diethyl ether anesthesia induces transient cytosolic [Ca^2+^] increase, heat shock proteins, and heat stress tolerance of photosystem II in *Arabidopsis*

**DOI:** 10.3389/fpls.2022.995001

**Published:** 2022-09-12

**Authors:** Andrej Pavlovič, Jana Jakšová, Zuzana Kučerová, Martina Špundová, Marek Rác, Pavel Roudnický, Axel Mithöfer

**Affiliations:** ^1^Department of Biophysics, Faculty of Science, Palacký University, Olomouc, Czechia; ^2^Central European Institute of Technology, Masaryk University, Brno, Czechia; ^3^Research Group Plant Defense Physiology, Max Planck Institute for Chemical Ecology, Jena, Germany

**Keywords:** anesthesia, *Arabidopsis*, chlorophyll, diethyl ether, heat shock proteins, heat stress, photosystem II

## Abstract

General volatile anesthetic diethyl ether blocks sensation and responsive behavior not only in animals but also in plants. Here, using a combination of RNA-seq and proteomic LC–MS/MS analyses, we investigated the effect of anesthetic diethyl ether on gene expression and downstream consequences in plant *Arabidopsis thaliana*. Differential expression analyses revealed reprogramming of gene expression under anesthesia: 6,168 genes were upregulated, 6,310 genes were downregulated, while 9,914 genes were not affected in comparison with control plants. On the protein level, out of 5,150 proteins identified, 393 were significantly upregulated and 227 were significantly downregulated. Among the highest significantly downregulated processes in etherized plants were chlorophyll/tetrapyrrole biosynthesis and photosynthesis. However, measurements of chlorophyll *a* fluorescence did not show inhibition of electron transport through photosystem II. The most significantly upregulated process was the response to heat stress (mainly heat shock proteins, HSPs). Using transgenic *A. thaliana* expressing *APOAEQUORIN*, we showed transient increase of cytoplasmic calcium level [Ca^2+^]_cyt_ in response to diethyl ether application. In addition, cell membrane permeability for ions also increased under anesthesia. The plants pre-treated with diethyl ether, and thus with induced HSPs, had increased tolerance of photosystem II to subsequent heat stress through the process known as cross-tolerance or priming. All these data indicate that diethyl ether anesthesia may partially mimic heat stress in plants through the effect on plasma membrane.

## Introduction

General volatile anesthetics (GVAs) are usually defined as compounds, which induce reversible loss of consciousness in humans (Franks, 2006). The clinical definition of anesthesiology states that it is the practice of medicine providing insensibility to pain during surgical, obstetric, therapeutic, and diagnostic procedures. Diethyl ether and chloroform were used as sole agents in a general anesthetic procedure for almost a century, and the term anesthesia was introduced soon after the discovery of etherization. In fact, the term anesthesia was coined to describe what happens during the process of etherization. GVAs produce unconsciousness, analgesia, amnesia, immobility, and lack of stress and hemodynamic responses in response to noxious stimulation (Urban and Bleckwenn, 2002). However, such narrow definitions are applicable only for subset of organisms with cortical networks that are susceptible to being anesthetized. Because the anesthetic drugs are also effective in organisms from protists, through plants, to primate, Kelz and Mashour (2019) proposed new definition for anesthetics applicable across whole tree of life as compounds which cause disconnection from environment, both in receptive (e.g., sensation) and expressive (e.g., motoric responses) arms of interaction.

If plants are exposed to GVAs, they indeed lost ability to sense their environment. In previous studies, it was shown that plants exposed to diethyl ether anesthesia were neither able to sense mechanical stimuli, wounding, or light and lack also expressive motoric responses. For example, touch-induced leaf movement in sensitive plant *Mimosa pudica*, trap closing reactions in carnivorous plant *Dionaea muscipula*, trap bending movement in carnivorous sundew *Drosera capensis* and autonomous circumnutations movements of tendrils of pea (*Pisum sativum*) were completely stopped (Milne and Beamish, 1999; De Luccia, 2012; Yokawa et al., 2018; Pavlovič et al., 2020; Böhm and Scherzer, 2021; Scherzer et al., 2022). We found that disappearance of some of these plant reactions were caused by inhibition of electrical signal generation and propagation, a target of anesthetics is remarkably similar to animal organisms. Also in the case of *Arabidopsis thaliana* plants, in which motoric responses are not easily observable, etherized individuals lost ability of systemic electrical and Ca^2+^ signals propagation from damaged to neighboring leaves after heat wounding (Jakšová et al., 2021). Since electrical and Ca^2+^ signal propagation is dependent on ligand-gated glutamate receptor like channels (GLRs, Mousavi et al., 2013; Toyota et al., 2018), and diethyl ether attenuated also glutamate-induced Ca^2+^ signal (Jakšová et al., 2021; Scherzer et al., 2022), GLR channels have been suspected as the possible targets of anesthesia in plants, like in animals. In the absence of electrical signals in etherized plants, the downstream sequence of events in systemic leaves were blocked, including accumulation of phytohormones of the jasmonates (JA) group and expression of JA-responsive genes, indicating the inhibition of sensing as well as responsive behavior in plants (Pavlovič et al., 2020; Jakšová et al., 2021).

The exact mode of GVA action in animals and plants is still a mystery. In the membrane theory, Meyer (1899) and Overton (1901) assumed that solubilization of lipophilic GVA in lipid bilayer of the neurons causes their fluidizing and malfunction, and anesthetic effect. In the modern lipid hypothesis, anesthetics do not act directly through the membrane, but rather perturb specialized lipid matrices at the protein-lipid interface (Lerner, 1997; Pavel et al., 2020). The protein theory of GVA action was put forward, when Franks and Lieb (1984) demonstrated that the anesthetic effect can be reproduced on a soluble luciferase protein in the absence of lipids. It was believed that GVAs bind to their target ion channel by a key-lock mechanism and change their structure dramatically from open to closed conformation. The modern protein theory suggests that GVAs do not change structure of membrane channel but change its dynamics, especially dynamics in the flexible loops that connect α-helices in a bundle and are exposed to the membrane-water interface (Tang and Xu, 2002). Recent findings indicate that GVAs disrupt lipid rafts, regions of ordered lipids which allow nanoscale compartmentalization of proteins and lipids (Pavel et al., 2020).

In this study, we focused on molecular responses to diethyl ether anesthesia in *A. thaliana* using transcriptomic (RNA-seq) and proteomic (LC–MS/MS) analyses. Although recent studies have shown that diethyl ether anesthesia blocks sensation and responsive behavior in plants, here we show for the first time that it also induced huge reprogramming of gene expression. Our data strongly suggest that GVA diethyl ether mimics a heat stress probably through the effect on plasma membrane.

## Materials and methods

### Plant material, culture conditions, and experimental setup

Plants of *Arabidopsis thaliana* (L.) Heynh. (Col-0) and transgenic *A. thaliana* (L.) Heynh. (Col-0), expressing the *APOAEQUORIN* gene under control of the CaMV 35S promoter, were grown on a soil substrate (Potgrond H, Klasmann-Deilmann, Germany) in a growth chamber (AR75L; Percival-Scientific, United States) for 6–7 weeks under 8 h light (100 μmol photons m^−2^ s^−1^ PAR)/16 h dark cycle (21/21°C) and 60% relative air humidity. The 6–7 weeks old plants were enclosed into polypropylene bags or transparent boxes and diethyl ether was applied. By adding a corresponding volume of liquid phase of diethyl ether to a certain volume of air, 15% vapor of diethyl ether was obtained (see Yokawa et al., 2018). Then, after 2.5 and 5.5 h, the leaf samples were collected and immediately frozen in liquid nitrogen and stored at −80°C. At the same time the leaf samples from controlled bagged plants without diethyl ether were also sampled by the same way.

### RNA-seq analyses

A single eighth leaf (for leaf numbering see Jakšová et al., 2021) of 2.5 h etherized and control plants of *A. thaliana* were cut, immediately frozen in liquid nitrogen and weighted. 50–60 mg of leaf material was homogenized in a Geno/Grinder® 2010 (Spex Sample Prep, Stanmore, United Kingdom) equipped with aluminum racks. The racks were cooled in liquid nitrogen prior to usage to prevent a thawing of leaf material during the whole homogenization process. RNA was extracted and purified using Trizol reagent (Invitrogen, Carlsbad, CA, United States) followed by the RNA Clean & Concentrator TM-5 kit (Zymo Research, Irvine, CA, United States), including DNase digestion to remove genomic DNA contamination. A total amount of 1 μg RNA per sample was used as input material for the RNA sample preparations. Sequencing libraries were generated using NEBNext® UltraTM RNA Library Prep Kit for Illumina® (NEB, United States) following manufacturer’s recommendations and index codes were added to attribute sequences to each sample. Briefly, mRNA was purified from total RNA using poly-T oligo-attached magnetic beads. Fragmentation was carried out using divalent cations under elevated temperature in NEBNext First Strand Synthesis Reaction Buffer (5X). First strand cDNA was synthesized using random hexamer primer and M-MuLV Reverse Transcriptase (RNase H-). Second strand cDNA synthesis was subsequently performed using DNA Polymerase I and RNase H. Remaining overhangs were converted into blunt ends *via* exonuclease/polymerase activities. After adenylation of 3′ ends of DNA fragments, NEBNext Adaptor with hairpin loop structure were ligated to prepare for hybridization. In order to select cDNA fragments of preferentially 150–200 bp in length, the library fragments were purified with AMPure XP system (Beckman Coulter, Beverly, United States). Then 3 μl USER Enzyme (NEB, United States) was used with size-selected, adaptor ligated cDNA at 37°C for 15 min followed by 5 min at 95°C before PCR. Then PCR was performed with Phusion High-Fidelity DNA polymerase, Universal PCR primers and Index (X) Primer. At last, PCR products were purified (AMPure XP system) and library quality was assessed on the Agilent Bioanalyzer 2100 system. The clustering of the index-coded samples was performed on a cBot Cluster Generation System using PE Cluster Kit cBot-HS (Illumina) according to the manufacturer’s instructions. After cluster generation, the library preparations were sequenced on an Illumina platform and paired-end reads were generated. Raw data (raw reads) of FASTQ format were firstly processed through FASTP. In this step, clean data (clean reads) were obtained by removing reads containing adapter and poly-N sequences and reads with low quality from raw data. At the same time, Q20, Q30, and GC content of the clean data were calculated. All the downstream analyses were based on the clean data with high quality. Differential expression analysis between two conditions/groups four biological replicates per condition was performed using DESeq2R package. DESeq2 provides statistical routines for determining differential expression in digital gene expression data using a model based on the negative binomial distribution. The resulting *p* values were adjusted using the Benjamini and Hochberg’s approach for controlling the False Discovery Rate (FDR). Genes with an adjusted *p* value <0.05 found by DESeq2 were assigned as differentially expressed. Gene Ontology (GO) enrichment analysis of differentially expressed genes was implemented by the clusterProfiler R package, in which gene length bias was corrected. GO terms with corrected *p* value less than 0.05 were considered significantly enriched by differential expressed genes. The RNA-seq experiment was commercially done by NovoGene.

### LC–MS/MS analyses

A single eight leaf from etherized *A. thaliana* plants for 5.5 h and non-etherized leaf from control plants were homogenized by mortar and pestle in liquid nitrogen after diethyl ether treatment. Homogenates were then lysed in SDT buffer (4% SDS, 0.1 M DTT, 0.1 M Tris/HCl, and pH 7.6) in a thermomixer (Eppendorf ThermoMixer® C, 60 min, 95°C, 750 rpm). After that, samples were centrifuged (15 min, 20,000 × *g*) and the supernatants (ca 100 μg of total protein) used for filter-aided sample preparation (FASP) as described elsewhere (Wiśniewski et al., 2009) using 0.75 μg of trypsin (sequencing grade; Promega). Resulting peptides were analyzed by LC–MS/MS.

LC–MS/MS analyses of all peptides were done using nanoElute system (Bruker) connected to timsTOF Pro spectrometer (Bruker). Two column (trapping column: Acclaim™ PepMap™ 100 C18, dimensions 300 μm ID, 5 mm long, 5 μm particles, Thermo Fisher Scientific; separation column: Aurora C18, 75 μm ID, 250 mm long, 1.6 μm particles, IonOpticks) mode was used on nanoElute system with default equilibration conditions (trap column: 10 volumes at 217.5 bars; separation column: 4 column volumes at 800 bars). Sample loading was done using three pickup volumes +2 μl at 100 bars. Trapped peptides were eluted by 120 min linear gradient program (flow rate 400 nl min^−1^, 3–80% of mobile phase B; mobile phase A: 0.1% FA in water; and mobile phase B: 0.1% FA in ACN). The analytical column was placed inside the Column Toaster (40°C; Bruker) and its emitter side was installed into CaptiveSpray ion source (Bruker). MSn data were acquired in m/z range of 100–1,700 and 1/k0 range of 0.6–1.6 V × s × cm^−2^ using DDA-PASEF method acquiring 10 PASEF scans with scheduled target intensity of 20,000 and intensity threshold of 2,500. Active exclusion was set for 0.4 min with precursor reconsideration for 4× more intense precursors.

For data evaluation, we used MaxQuant software (v1.6.17; Cox and Mann, 2008) with in built Andromeda search engine (Cox et al., 2011). Search was done against protein databases of *A. thaliana* (27,468 protein sequences, version from 2020-12-02, downloaded from ftp://ftp.uniprot.org/pub/databases/uniprot/current_release/knowledgebase/reference_proteomes/Eukaryota/UP000006548/UP000006548_3702.fasta.gz) and cRAP contaminants (112 sequences, version from 2018-11-22, downloaded from http://www.thegpm.org/crap). Modifications were set as follows for database search: oxidation (M), deamidation (N, Q), and acetylation (Protein N-term) as variable modifications, with carbamidomethylation (C) as a fixed modification. Enzyme specificity was tryptic with two permissible miscleavages. Only peptides and proteins with false discovery rate threshold under 0.01 were considered. Relative protein abundance was assessed using protein intensities calculated by MaxQuant. Intensities of reported proteins were further evaluated using software container environment (https://github.com/OmicsWorkflows/KNIME_docker_vnc; version 4.1.3a). Processing workflow is available upon request and it covers, in short, reverse hits and contaminant protein groups (cRAP) removal, protein group intensities log2 transformation and normalization (loessF), and LIMMA statistical tests. Significantly up/downregulated protein groups were further subjected to functional enrichment analysis using g:Profiler web tool (significance threshold g:SCS; user threshold 0.05; Raudvere et al., 2019).

### Western blotting

Total protein from the eighth leaf of 5.5 h etherized and control plants of *A. thaliana* was isolated using extraction buffer containing 28 mM DTT, 28 mM Na_2_CO_3_, 175 mM sucrose, 5% SDS, 10 mM EDTA, and protease inhibitors (Set VI, Calbiochem, Darmstadt, Germany). The samples were heated for 30 min at 70°C. The concentration of total soluble proteins in the samples was determined using the Bicinchoninic Acid Kit for Protein Determination (Sigma-Aldrich, St. Louis, MO, United States), and absorbance was measured at 562 nm (Thermo Spectronic UV500, UV–Vis Spectro, MA, United States). The same amount of protein was separated in a 10% (v/v) SDS–polyacrylamide gel (Schägger, 2006) followed by transfer to a nitrocellulose membrane (Bio-Rad, Germany) by Trans-Blot SD Semi-Dry Electrophoretic Transfer Cell (Bio-Rad, Hercules, CA, United States). To check the correct protein transfer, the membranes were stained by Ponceau-S. After blocking in TBS-T containing 5% BSA overnight at 4°C, the membranes were incubated with the primary antibody at room temperature with soft agitation. Antibodies against proteins HSP70 (AS08371), HSP90-1 (AS08346), GluTR (AS10689), LPOR (AS05067), RbcL (AS03037), and actin (AS13 2640) were purchased from Agrisera (Vännäs, Sweden). After washing, the membrane was incubated 1 h with the secondary antibody [goat anti-rabbit IgG (H + L)-horseradish peroxidase conjugate] with dilution 1:10,000 (Bio-Rad, Hercules, CA, United States). Signals were visualized and quantified using Immobilon Western chemiluminescent HRP substrate (Millipore, Billerica, MA, United States) on an Amersham Imager 600 (GE HealthCare Life Sciences, Japan). The data were checked for homogeneity of variance and significant differences were evaluated by Student’s *t*-test. If non-homogeneity was present, Welch’s *t*-test was used instead (Microsoft Excel).

### Aequorin luminescence imaging

Transgenic *A. thaliana* (L.) Heynh Col-0 wild-type expressing the *APOAEQUORIN* gene under control of the CaMV 35S promoter was used for [Ca^2+^]_cyt_ analyses (Kiep et al., 2015). Aequorin was reconstituted by spraying plants with 10 μM coelenterazine (Invitrogen, Eugene, OR, United States) in 0.01% Tween 20 (Sigma-Aldrich, United Kingdom) and subsequent incubation for 5 h in the dark. Aequorin luminescence imaging was performed using a highly sensitive CCD camera iKon-XL (Oxford Instruments plc, Tubney Woods, Abingdon, United Kingdom). To reduce the dark current, CCD camera was cooled down to −100°C. The CCD camera was equipped with a 50-mm focal distance lens with an f-number of 1.2 (Nikon, Tokyo, Japan) to enhance the light collecting efficiency. Spectral sensitivity of CCD camera was within the range of λ = 200–1,000 nm with almost 90% quantum efficiency in the visible range of the spectrum. The spectral sensitivity was limited to λ = 350–1,000 nm by the lenses. CCD camera parameters were as follows: scan rate, 100 kHz; gain, 2. Photons were captured in photon-counting mode with a 5 min acquisition time. Signal acquisition and processing were performed with Andor Solis (Oxford Instruments plc, Tubney Wood, Abingdon, United Kingdom) and ImageJ 1.49 (NIH, United States), respectively. The CCD camera was situated in the experimental dark room (3 m × 1.5 m × 2.5 m) painted in black. The door in the experimental dark room was protected completely with a black curtain to restrict any external light. The plants were imaged 10 min before, during and 2.5 h after diethyl ether application. All experiments were repeated several times to ensure reproducibility.

### Chlorophyll *a* fluorescence quenching analysis

Chlorophyll *a* fluorescence quenching analysis was measured using a FluorCam imaging system (800–0, PSI, Czech Republic) on the plants enclosed in the transparent boxes. The plants were treated by diethyl ether for 5.5 h and measured immediately still under the effect of diethyl ether. Control plants were also enclosed in the box before and during the measurement in order to maintain the same optical conditions of the measurement. Before measurements, the plants were dark-adapted for 20 min. A kinetics of three parameters—an effective quantum yield of photosystem II (PSII; Φ_PSII_), excitation pressure on PSII (1–q_P_), and non-photochemical quenching of chlorophyll fluorescence (NPQ) were evaluated after switching on actinic light (red light, 100 μmol photons m^−2^ s^−1^ PAR) and during subsequent dark relaxation. Φ_PSII_ was calculated as Φ_PSII_ = (F_M_´ – F_t_)/F_M_´, the excitation pressure 1–q_P_ = 1 – (F_M_´ – F_t_)/(F_M_´ – F_0_´), and NPQ = (F_M_ – F_M_´)/F_M_´ (Maxwell and Johnson, 2000). Maximum fluorescence in dark (F_M_) and light-adapted state (F_M_´) was determined by applying the 1.6 s saturating pulse (blue light, 3,000 μmol photons m^−2^ s^−1^ PAR). The actual fluorescence signal at the time *t* of actinic illumination (F_t_) was measured immediately prior to the application of saturating pulse. Minimum fluorescence in light-adapted state (F_0_´) was estimated by the formula F_0_´ ≈ F_0_ /[(F_M_ – F_0_)/F_M_ + F_0_/F_M_´]. F_0_ (minimum fluorescence in dark-adapted state) was determined by applying several μ-seconds-long measuring flashes (red light, 0.1 μmol photons m^−2^ s^−1^ PAR) at the beginning of the procedure. The data were checked for homogeneity of variance and significant differences were evaluated by Student’s *t*-test, if non-homogeneity was present, Welch’s *t*-test was used instead (Microsoft Excel).

### Measurements of fast chlorophyll *a* fluorescence transient

To investigate the effect of diethyl ether mediated cross-tolerance or priming on subsequent heat–stress response, we measured fast chlorophyll *a* fluorescence transient. The measurements were done in control plants and plants incubated 5.5 h in diethyl ether. Then the plants were removed from the boxes and after 30 min of their recovery (15 min under dim light and then 15 min in darkness), leaves were detached from the plants and incubated for 5 min in a water bath at room temperature (RT) or at 40, 42, 43, 45, and 46°C (in darkness). Immediately after the heat treatment, chlorophyll *a* fluorescence induction transient (OJIP curve) was measured by a Plant Efficiency Analyzer (Hansatech Instruments, United Kingdom) at RT from the adaxial side of the leaves. Excitation light intensity of 2,500 μmol photons m^−2^ s^−1^ PAR (red light) and 2 s detection time were used for the measurement. A maximal quantum yield of PSII photochemistry was estimated as F_V_/F_M_ = (F_M_ – F_0_)/F_M_, where F_M_ is maximal fluorescence (corresponding to the fluorescence intensity in P-level in the OJIP curve) and F_0_ minimal fluorescence in dark-adapted leaves (Maxwell and Johnson, 2000). The measured O(K)JIP curves were normalized to variable fluorescence (F_V_ = F_M_ – F_0_) and to variable fluorescence at 2 ms [F_V(2ms)_ = F_(2ms)_ – F_0_] in order to better visualize a K-band indicating high temperature-induced inhibition of oxygen evolving complex (Guissé et al., 1995; Srivastava et al., 1997). The data were checked for homogeneity of variance and significant differences were evaluated by Student’s *t*-test, if non-homogeneity was present, Welch’s *t*-test was used instead (Microsoft Excel).

### Measurements of cell membrane permeability for ions

For the determination of the extent of ion leakage from leaf tissue, as a measure of cell membrane permeability for ions associated with membrane damage and/or increased fluidity, leaf disks (diameter of 12 mm) were cut out from leaves of the control plants and plants, which had been incubated in diethyl ether for 5.5 h before. Three leaf disks (representing one sample) were immediately put into test tube containing 5 ml of deionized water and incubated in water bath at RT or temperature of 42 or 45°C. The electrical conductivity (EC) of the solutions was measured in 10 min intervals (at RT) or after 60 min of incubation of the samples at a given temperature with a digital conductivity meter (GMH 3430, Greisinger, Germany). The total electrical conductivity (EC_T_) was measured after autoclaving the samples for 15 min at 121°C. The relative conductivity (%) was calculated as EC/EC_T_ 100. The data were checked for homogeneity of variance and significant differences were evaluated by Student’s *t*-test, if non-homogeneity was present, Welch’s *t*-test was used instead (Microsoft Excel).

## Results

### Transcriptomic analyses

**Figure 1:**
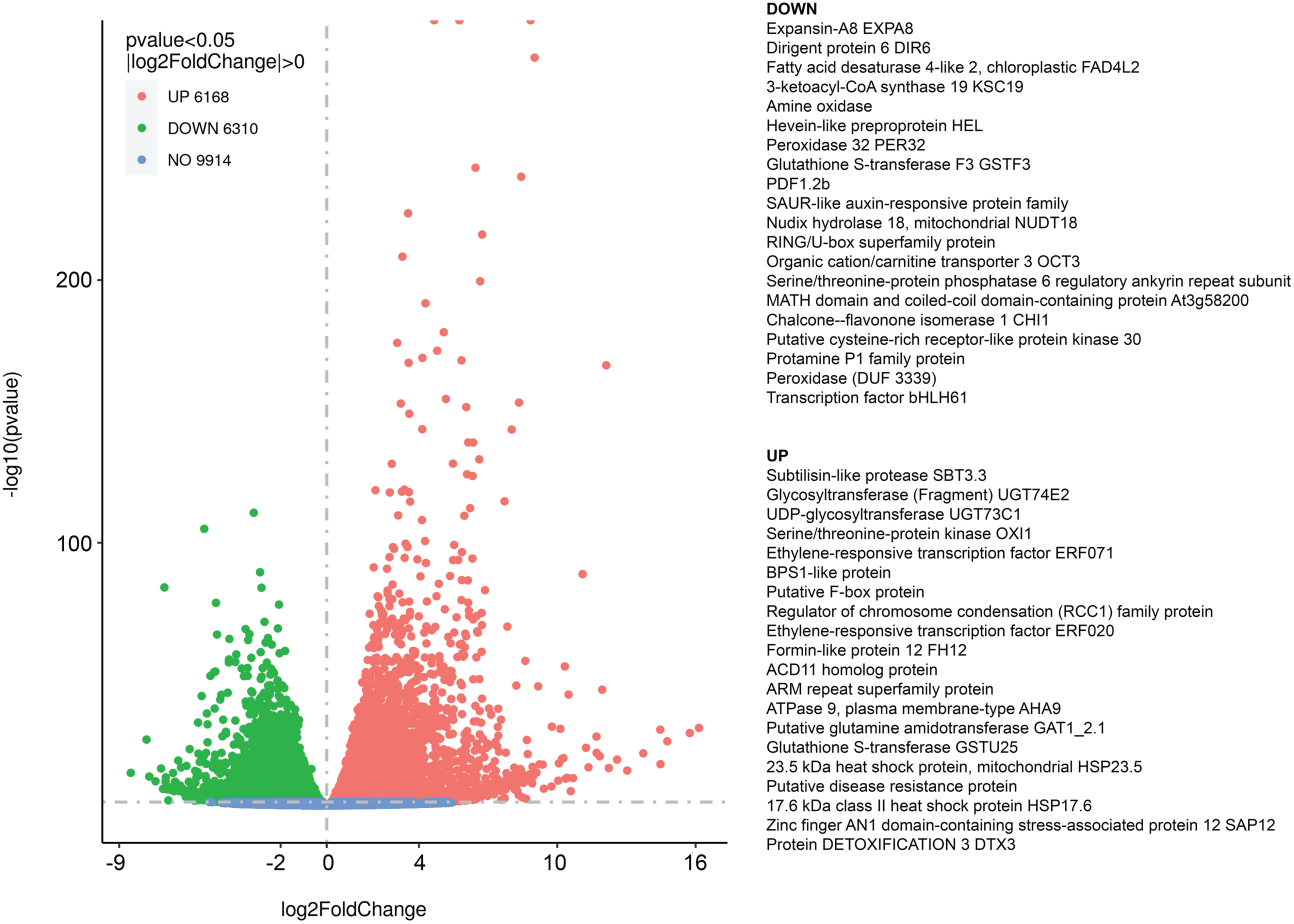
Summary of the RNA-seq results and differentially expressed genes (DEG) in response to diethyl ether anesthesia in *Arabidopsis thaliana*. Volcano plot representation of differential expression analysis of genes in the control *vs*. diethyl ether treated plants. Green and red points mark the genes with significantly decreased or increased expression, respectively in diethyl ether treated plants compared to controls (*p* < 0.05). Blue points mark the genes that are not significantly differentially expressed. The *x*-axis shows log_2_ fold changes in expression and the *y*-axis the log_10_ *p* values of a gene being differentially expressed. On the right, top 20 upregulated and top 20 downregulated genes based on log_2_ fold change are listed. The complete list of DEG is available as Supplementary Table S1.

**Figure 2:**
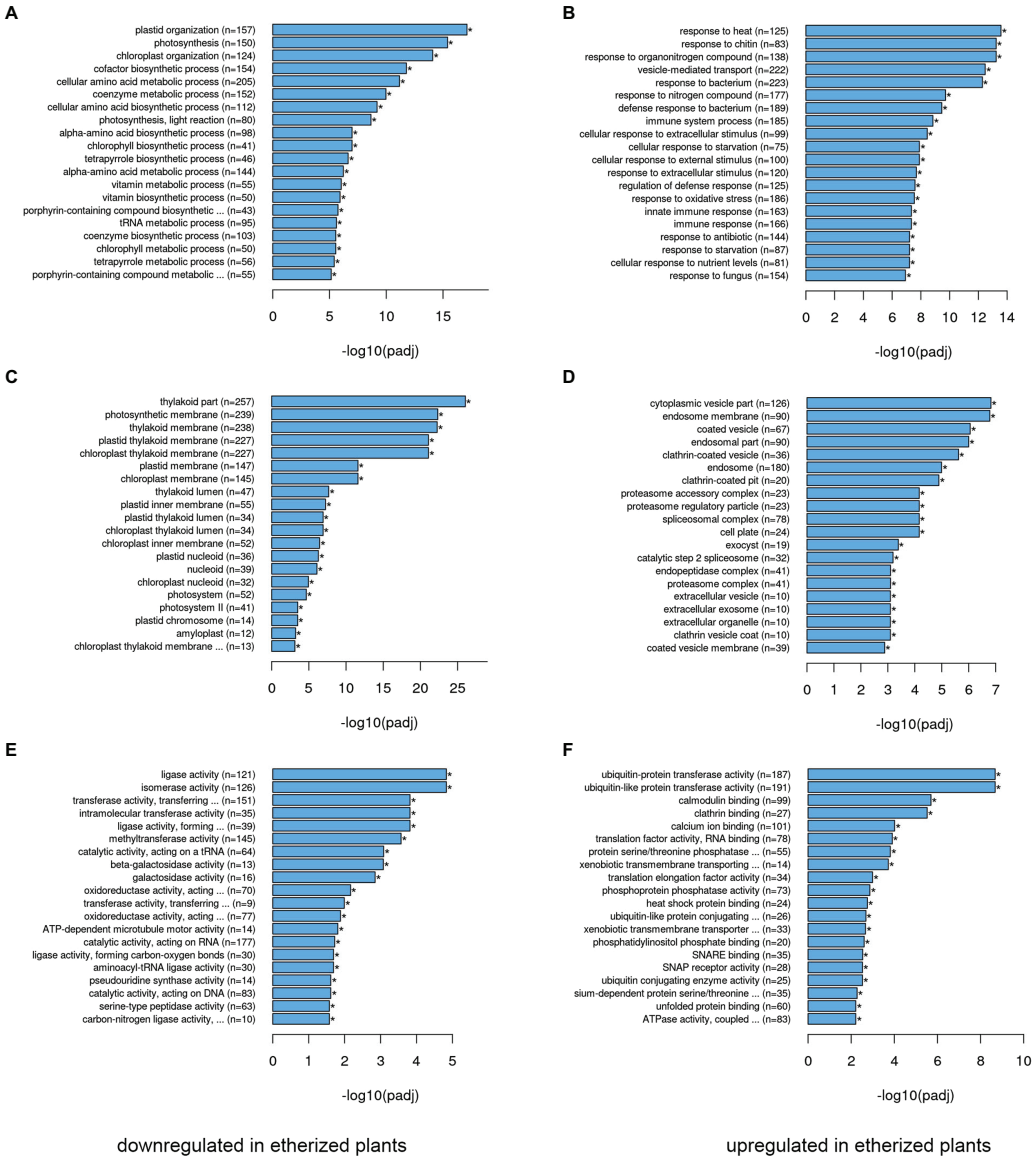
Gene ontology (GO) functional analysis of differentially expressed genes (DEGs) in *Arabidopsis thaliana*. The 20 most significantly (*p* < 0.05) enriched downregulated GO terms in biological process **(A)**, cellular component **(C)**, and molecular function **(E)** branches in etherized plants compared to control are presented. The 20 most significantly (*p* < 0.05) enriched upregulated GO terms in biological process **(B)**, cellular component **(D)**, and molecular function **(F)** branches in etherized plants compared to control are presented. All the adjusted statistically significant values of the terms were negative 10-base log transformed. Asterisks (*) denote significantly different GO terms at *p* < 0.05. A complete list of GO terms is available as Supplementary Table S2.

We performed transcriptomic studies with *A. thaliana* plants that were exposed to diethyl ether anesthesia for 2.5 h. Differential expression analyses revealed that under anesthesia 9,914 genes were not affected, 6,168 genes were upregulated and 6,310 genes were downregulated at *p* < 0.05 in comparison to control plants (Figure 1). The complex lists of upregulated and downregulated genes are available in Supplementary Table S1. For annotation and categorization a gene product’s molecular function (MF), cell compartment (CC) and associated biological process (BP) gene ontology enrichment analyses (GO) were performed (Figure 2). Among the top 20 downregulated processes in etherized plants were biological processes (GO-BP) involved in photosynthesis, chlorophyll/tetrapyrrole biosynthesis, amino acids metabolism, and cofactor/coenzyme biosynthesis (Figure 2A). Not surprisingly, the locations relative to cellular structures in which the downregulated gene products perform a function (GO-CC) were chloroplasts and thylakoid membranes (Figure 2C). Molecular function activities (GO-MF) involved ligase, isomerase, transferase, oxidoreductase, and galactosidase activities (Figure 2E). Among the top 20 upregulated processes in etherized plants were biological processes (GO-BP) involved in heat response, response to chitin and bacterium, vesicle-mediated transport, immune system responses, response to (organo)nitrogen compounds etc. (Figure 2B). The locations where the upregulated genes perform their function (GO-CC) were mainly in endomembrane system (Figure 2D). The upregulated molecular functions (GO-MF) were represented by protein degradation (ubiquitin system), modification (phosphatases), folding (heat shock protein binding), vesicular transport (clathrin and SNARE binding), and calcium ion and calmodulin binding (Figure 2F). The complex lists of significantly enriched GO terms are available in Supplementary Table S2.

### Proteomic analysis

**Figure 3:**
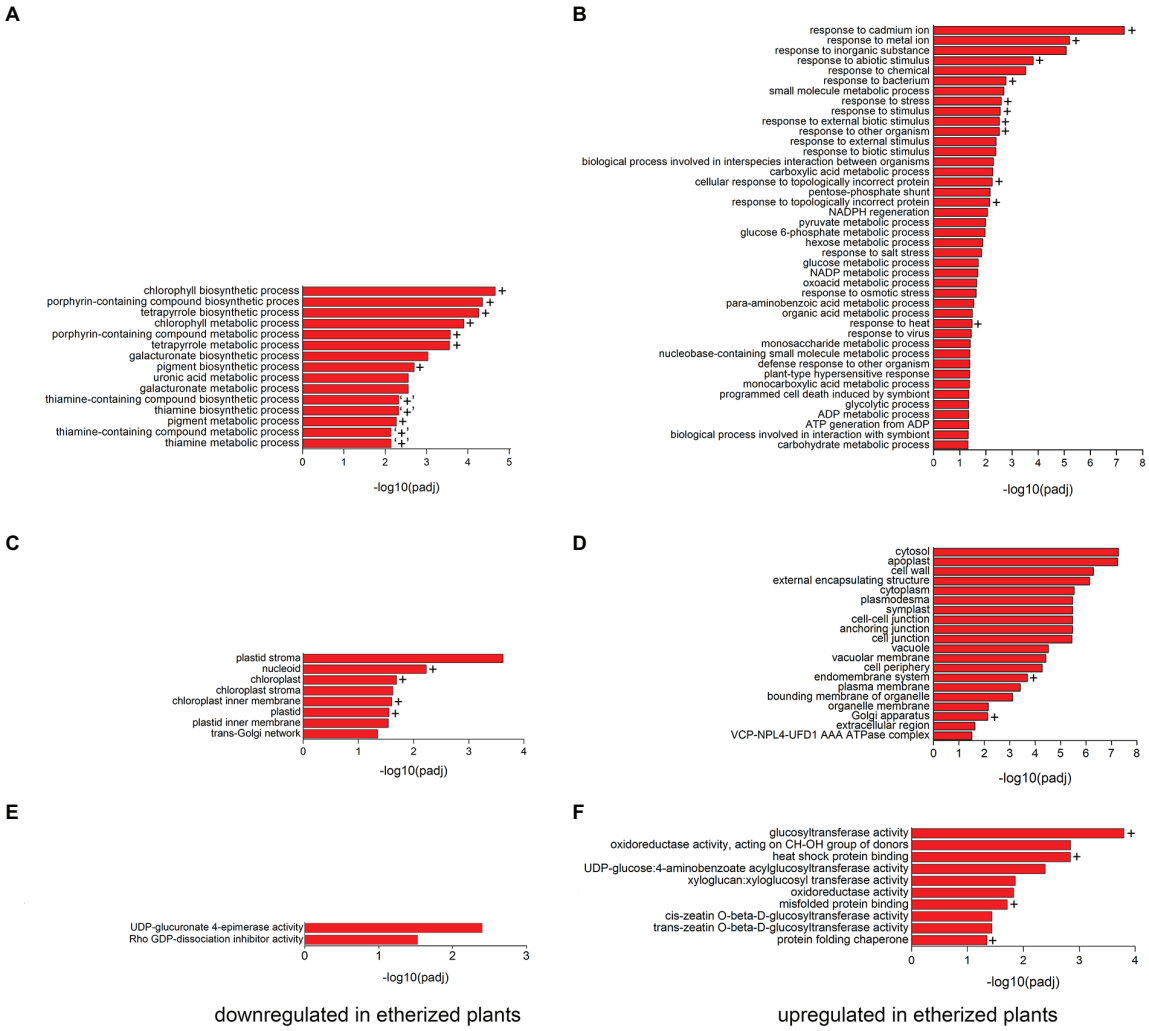
Gene ontology (GO) functional analysis of differentially expressed proteins (DEPs) in *Arabidopsis thaliana*. All the significantly (*p* < 0.05) enriched downregulated GO terms in biological process **(A)**, cellular component **(C)**, and molecular function **(E)** branches in etherized plants compared to control are presented. All the significantly (*p* < 0.05) enriched upregulated GO terms in biological process **(B)**, cellular component **(D)**, and molecular function **(F)** branches in etherized plants compared to control are presented. All the adjusted statistically significant values of the terms were negative 10-base log transformed. Crosses (+) denote the same or very similar GO terms, which were enriched also in RNA-seq experiments. “+” denote similar GO term from RNA-seq experiments (vitamin metabolic and vitamin biosynthetic processes) which contain downregulated genes for thiamine biosynthesis. A complete list of DEP is available as Supplementary Table S3.

To validate the RNA-seq data and to confirm if increased/decreased level of mRNA is mirrored also on protein level, we performed proteomic analysis of plants etherized for 5.5 h. This longer treatment was chosen based on the fact that proteins have slower turnover rate than mRNA. Out of 5,150 proteins detected and identified, 393 proteins were significantly upregulated, and 227 proteins were significantly downregulated at *p* < 0.05 in etherized plants in comparison to control plants. The list of upregulated and downregulated proteins is available as Supplementary Table S3. As in the case of RNA-seq experiment, GO analysis was performed (Figure 3). Among the significantly downregulated processes in etherized plants were biological processes involved in chlorophyll/tetrapyrrole biosynthesis, uronic acid and galacturonate metabolic processes and thiamine metabolism (GO-BP, Figure 3A), which were located in different parts of plastid/chloroplast and Golgi network (GO-CC, Figure 3C). UDP-glucuronate 4-epimerase activity and Rho GDP-dissociation inhibitor activity were the only two significantly downregulated molecular functions (GO-MF, Figure 3E). Among the significantly upregulated processes in etherized plants were biological processes involved in response to cadmium/metal ions, responses to different biotic/abiotic stimuli, response to heat etc. (GO-BP, Figure 3B) in different cell parts (Figure 3D). The upregulated molecular functions (GO-MF) involved glucosyltransferase activity, xyloglucan/xyloglucosyl transferase activity and *cis*/*trans* zeatin O-beta-D-glucosyltransferase activity, protein folding (heat-shock protein binding, misfolded protein binding, and protein folding chaperone) etc. (GO-MF, Figure 3F).

### Anesthesia with diethyl ether downregulated chlorophyll metabolism and upregulated heat shock proteins

**Figure 4:**
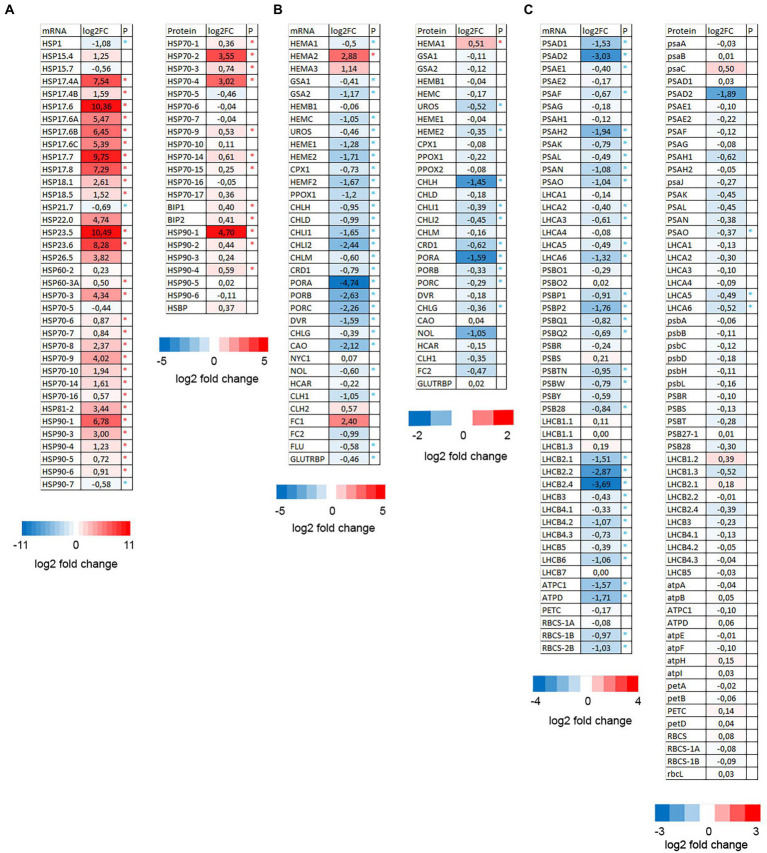
Heat maps showing the expression pattern of HSPs **(A)**, chlorophyll metabolism **(B)**, and photosynthesis-related genes **(C)** in *Arabidopsis thaliana*. Log_2_ fold changes in the mRNA (from RNA-seq experiment) and proteins (LC–MS/MS analysis) in diethyl ether treated plants compared to controls (asterisk denote significant differences at *p* < 0.05, *n* = 4).

When comparing RNA-seq experiments and proteomic analyses one might notice that anesthesia with diethyl ether upregulated genes and proteins involved in reparation of misfolded proteins (mainly HSPs) and downregulated photosynthesis/chlorophyll metabolism. Among other processes were upregulation of endomembrane/vesicular transport associated genes and downregulated vitamin/thiamine metabolic processes. Indeed heat maps indicate that majority of HSPs mRNA/proteins were significantly upregulated and mRNA/proteins involved in chlorophyll metabolism were significantly downregulated (Figure 4). The proteins involved in photosynthesis were significantly downregulated only in three cases (PsaO, Lhca5, and Lhca6) despite significant transcriptional downregulation of many genes involved in photosynthesis (Figure 4). This was probably caused by the high abundance of majority of photosynthesis-related proteins which accumulated 6–7 weeks before short diethyl ether treatment (5.5 h) what was a too short time to reverse the accumulation. This is in line with chlorophyll concentration which did not differ significantly between control and etherized plants (data not shown).

**Figure 5:**
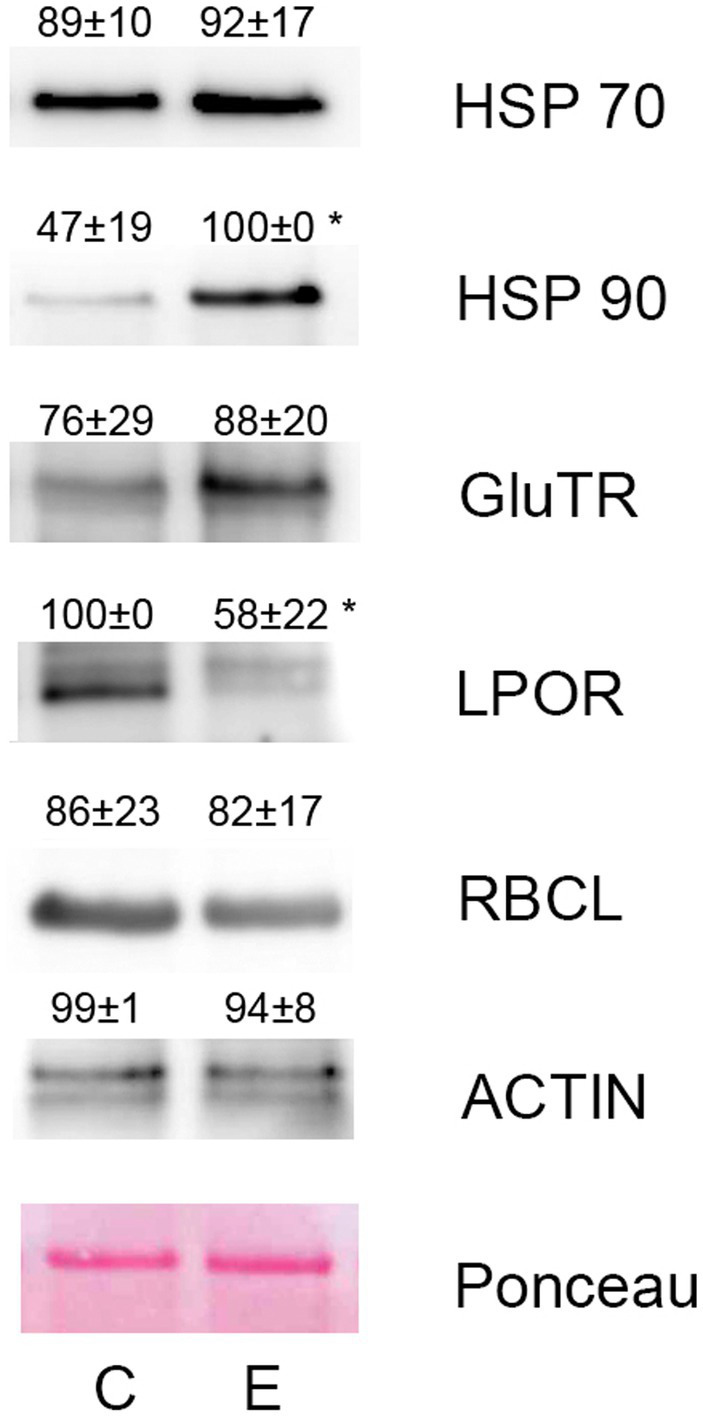
Western blotting of selected proteins involved in heat stress response and chlorophyll metabolism in *Arabidopsis thaliana*. The same protein amount was separated in 10% (v/v) SDS-PAGE and subjected to Western blot analysis. Antibodies against HSP70, HSP90, GluTR, LPOR, RbcL, and actin were used. The representative blots from three independent isolations are shown. The quantification of chemiluminescence signal is shown above the corresponding band. Means ± SD. C, control plants; E, etherized plants. Asterisks (*) denote significant differences at *p* < 0.05 (Student’s or Welch’s *t*-test), *n* = 3.

Western blotting using commercial antibodies reacting with different isoforms of heat shock proteins HSP70 and HSP90 showed only negligible increase of HSP70 isoforms (reacting with HSP70-1, HSP70-2, and HSP70-3) in response to diethyl ether treatment, however antibodies against HSP90 reacting with HSP90-1 and HSP90-2 showed significant increase in etherized plants. The key regulatory enzymes in chlorophyll biosynthesis glutamyl-tRNA reductase (GluTR or HEMA) and light-dependent protochlorophyllide oxidoreductase (POR) were also immunoblotted. The antibody against GluTR reacting with two isoforms (HEMA1 and HEMA2) in *Arabidopsis* showed slightly higher enzyme abundance in etherized plants but not significant. On the contrary, POR was significantly less abundant in etherized plants (Figure 5). The antibody against large subunit of Rubisco (RbcL) and actin were used as loading controls and their content was not affected by diethyl ether treatment as data from proteomic analyses indicates. Western blotting analyses confirmed RNA-seq and proteomic data.

### Diethyl ether induced calcium entry into the cells

**Figure 6:**
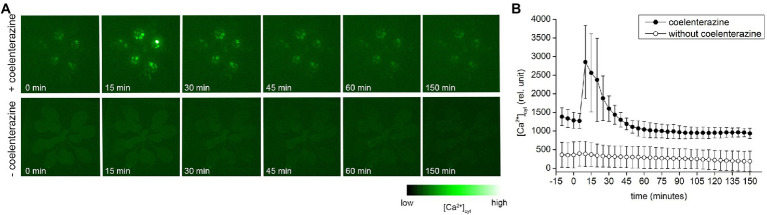
The cytoplasmic Ca^2+^ [Ca^2+^]_cyt_ signals in whole *Arabidopsis thaliana* rosette expressing *APOAEQUORIN* in response to diethyl ether application. **(A)** Representative images of selected time points of plants treated (upper row) and non-treated (lower row) with coelenterazine. **(B)** Time course (0–250 min) of average [Ca^2+^]_cyt_ accumulation in whole rosette in response to diethyl ether application. Plants treated with coelenterazine (closed circles) or without it (open circles). The diethyl ether was applied at time point 1 min. Means ± SD, *n* = 4. The time lapse video of [Ca^2+^]_cyt_ is available as Supplementary Videos S1, S2.

Because heat shock response and accumulation of HSPs were attributed to the entry of Ca^2+^ into the cytosol (Saidi et al., 2009), we measured [Ca^2+^]_cyt_ in transgenic *A. thaliana* plants expressing *APOAEQUORIN* treated with coelenterazine for AEQUORIN reconstitution, in response to diethyl ether application. Within few minutes after diethyl ether application, the rapid rise of [Ca^2+^]_cyt_ signal was detected and the signal slowly decayed later (Figure 6; Supplementary Video S1). To prove that the signal is not an ultra-weak photon emission, the experiments were repeated also with transgenic plants without coelenterazine treatment. No clear signal in response to diethyl ether application was detected (Figure 6; Supplementary Video S2) confirming that the signal in AEQUORIN plants came from increased [Ca^2+^]_cyt_.

### Diethyl ether affected photosynthetic parameters in etherized plants

**Figure 7:**
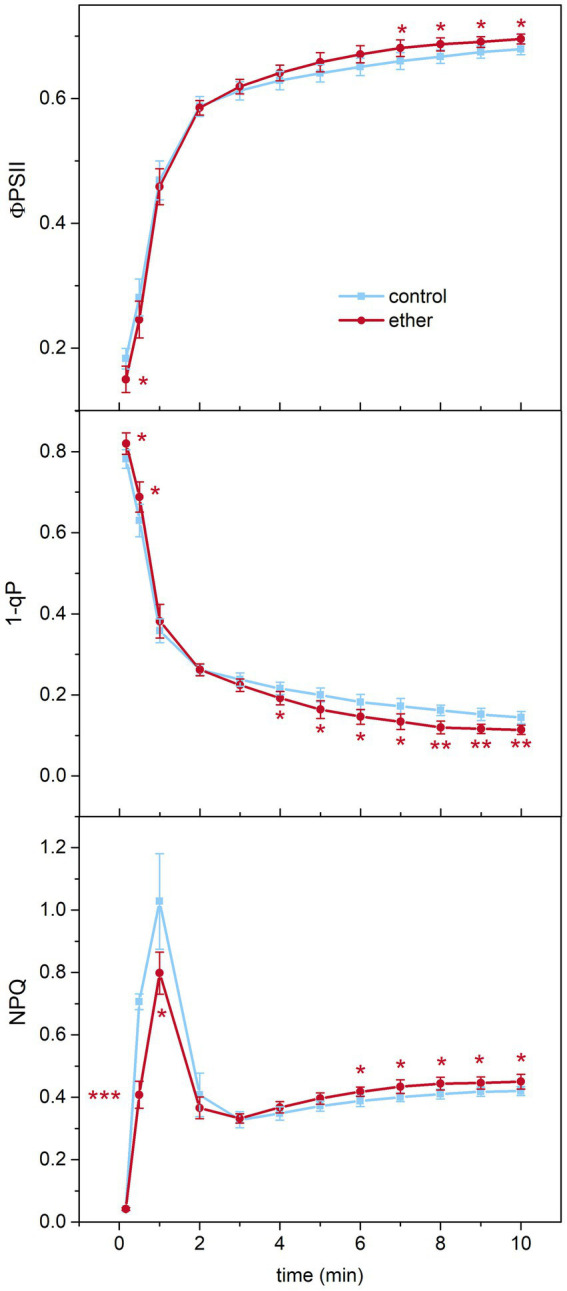
Chlorophyll *a* fluorescence parameters in *Arabidopsis thaliana*. A kinetics of effective photochemical quantum yield of PSII (Φ_PSII_), excitation pressure on PSII (1–qP), and non-photochemical quenching (NPQ) of chlorophyll *a* fluorescence after switching on actinic light (time point 0 min) in control and etherized plants. Asterisks denote significant difference at *p* < 0.01 (**) and *p* < 0.05 (*), (Student *t*-test). Means ± SD, *n* = 5 (Student’s *t*-test).

Due to the significant effect of diethyl ether on chlorophyll biosynthesis and photosynthesis, we investigated changes in chlorophyll *a* fluorescence parameters reflecting transition of photosynthetic apparatus from dark- to light-adapted state by measurements of chlorophyll *a* fluorescence quenching analysis. We found that etherized plants exhibited lower Ф_PSII_, higher 1-qP, and lower NPQ during the first 30 s of actinic illumination. Later, after steady-state conditions were achieved, higher Ф_PSII_, lower 1-qP, and higher NPQ were found in etherized plants (Figure 7).

### Diethyl ether anesthesia protected OEC and PSII against subsequent heat stress

**Figure 8:**
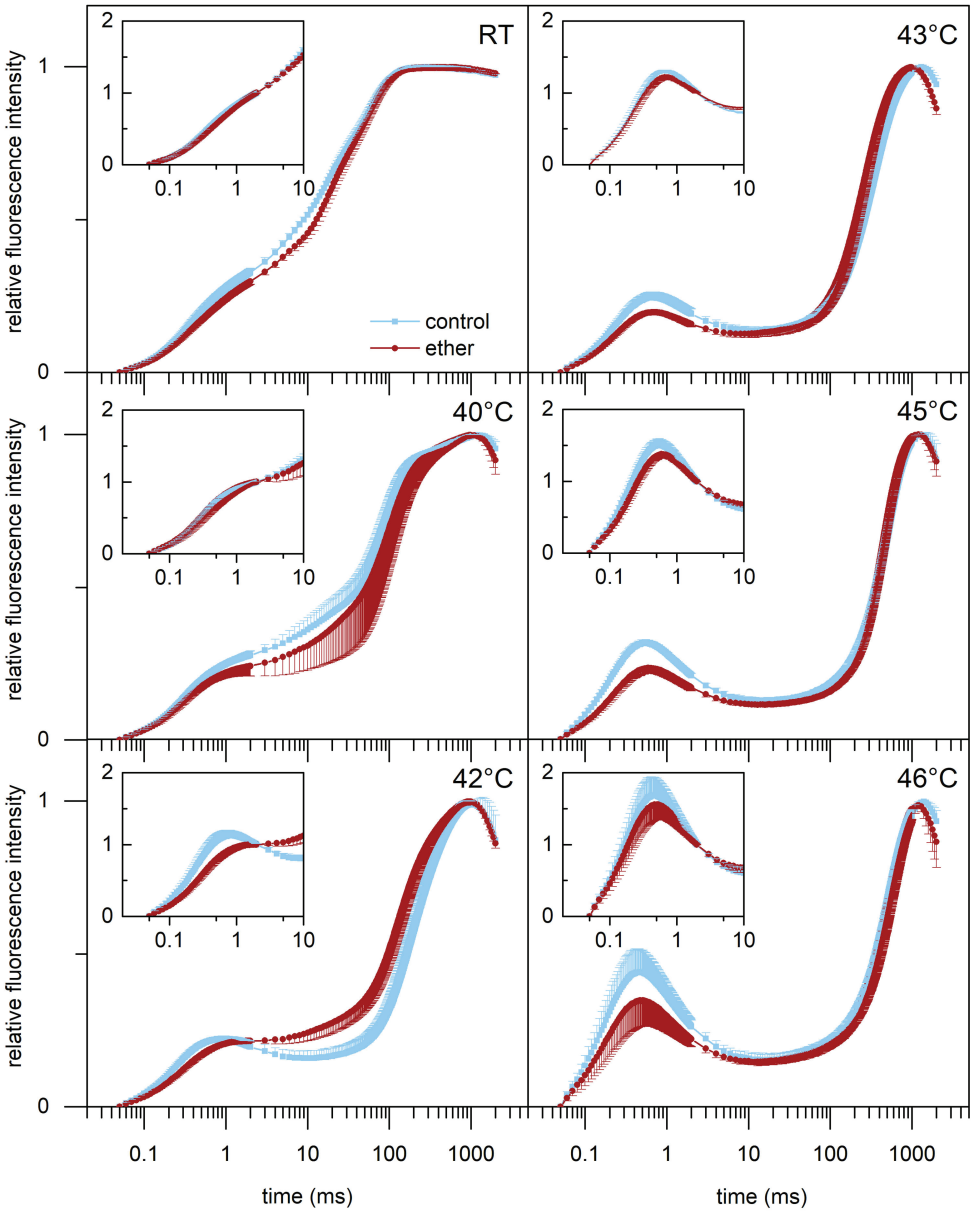
Effect of high-temperature treatment on chlorophyll *a* fluorescence induction transient in leaves of control and diethyl ether pre-treated *Arabidopsis thaliana* plants. Detached leaves were incubated for 5 min in a water bath of given temperature in darkness. RT, room temperature. The transients are normalized to variable fluorescence (F_V_ = F_M_ – F_0_) or to variable fluorescence at 2 ms [F_V(2ms)_ = F_(2ms)_ – F_0_; inlets]. Means ± SD, *n* = 5.

**Figure 9:**
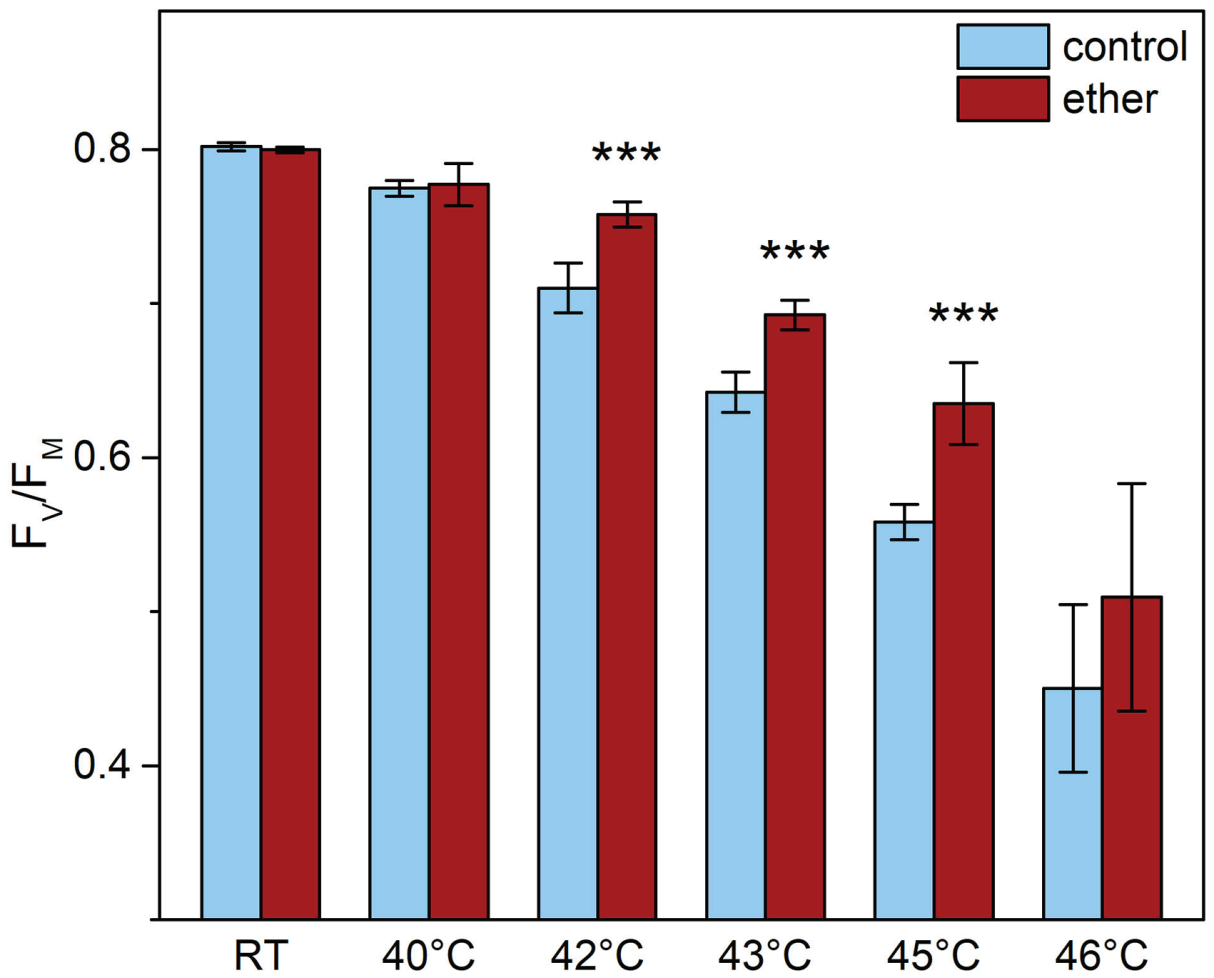
Maximal quantum yield of PSII photochemistry (F_V_/F_M_) in leaves of control and diethyl ether pre-treated *Arabidopsis thaliana* plants. Detached leaves were incubated for 5 min in a water bath of given temperature in darkness. RT, room temperature. Means ± SD, *n* = 5. Asterisks (***) indicate statistically significant difference from untreated samples (*p* < 0.001; Student’s or Welch’s *t*-test).

Because anesthesia induced HSPs, we decided to investigate a possible protective role of diethyl ether anesthesia against subsequent heat stress. Among the primary target of thermal damage in plants is the oxygen evolving complex (OEC) in PSII, which can be easily monitored by measurements of fast induction kinetics of chlorophyll *a* fluorescence. We monitored the appearance of K-step which is a sensitive indicator of OEC and PSII damage (Lazár et al., 1997; Srivastava et al., 1997) in response to increasing temperature. At room temperature, no clear K-step was detectable, but increasing the temperature above 40°C for 5 min resulted in appearance of K-step at about 0.5 ms (Figure 8) and decrease of maximum quantum yield of PSII (F_v_/F_m_, Figure 9). Both the less pronounced K-step and the significantly reduced decrease of F_v_/F_m_ upon increasing temperature in diethyl ether exposed plants indicate protective effects of diethyl ether anesthesia against high temperatures caused damage of OEC and PSII.

### Diethyl ether increased membrane permeability for ions in etherized plants at room temperature

**Figure 10:**
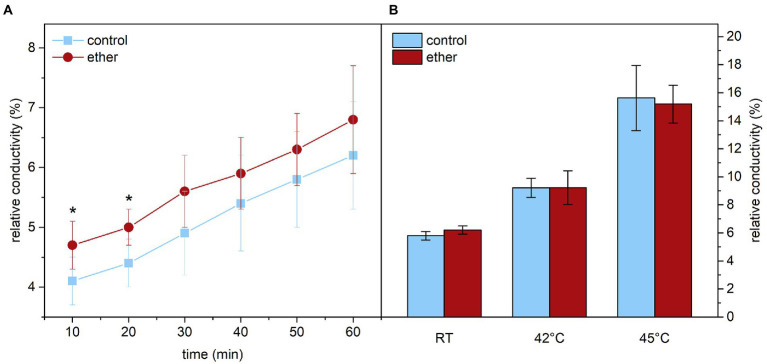
Relative conductivity reflecting ion leakage from leaf samples of *Arabidopsis thaliana*. **(A)** Relative conductivity at room temperature (RT) measured in 10 min intervals immediately after diethyl ether treatment and in control samples. **(B)** Relative conductivity after 60 min incubation of leaf samples at given temperature. Expressed in % of maximum conductivity measured in leaf samples with fully disintegrated membranes. Means ± SD, *n* = 5. Asterisks (*) indicate statistically significant difference from untreated samples (*p* < 0.05; Student’s or Welch’s *t*-test).

Because cell membranes including their permeability for ions are strongly affected by higher temperatures (Saidi et al., 2009), we next investigated the effect of diethyl ether on ion leakage from leaf samples. At room temperature, the relative electrical conductivity was slightly but significantly higher in etherized plants for the first 20 min of measurements indicating higher ion leakage (Figure 10A). Later, the difference between etherized and control plants were not significant probably due to longer measuring period and recovery from diethyl ether treatment. In an additional experiment, we compared the relative conductivity of leaves exposed for 60 min to RT, 42 and 45°C. Typically, the relative conductivity increased with increasing temperature reflecting an increase in membrane permeability for ions due to membrane changes not related to damage (leading probably to the efflux of K^+^ ions together with their counter ions, Demidchik et al., 2014; Ilík et al., 2018). Diethyl ether had no additional effect on the ion leakage at higher temperature (Figure 10B).

## Discussion

Recent studies showed that the anesthetic diethyl ether strongly suppresses the sensing of different stimuli in plants (e.g., light, touch, or wounding, Milne and Beamish, 1999; De Luccia, 2012; Yokawa et al., 2018; Pavlovič et al., 2020; Böhm and Scherzer, 2021; Jakšová et al., 2021; Scherzer et al., 2022). Here we show it concomitantly triggers also a strong cellular response. This omics-based study demonstrates that exposure of plants to GVA diethyl ether strongly reprogrammed gene expression in *A. thaliana*. Among the most obvious upregulated genes/proteins were HSPs (Figure 4), but also other heat responsive genes (e.g., WRKY transcription factors, Cheng et al., 2021). Studies on animals also found that GVA profoundly changed gene expression pattern (Sergeev et al., 2004) and increased expression of HSPs (HSP-10, HSP-27, and HSP70-1; Sergeev et al., 2004; Coghlan et al., 2018; Upton et al., 2020). HSPs are essential components contributing to cellular homeostasis under both optimal and detrimental conditions and are responsible for protein folding, assembly, translocation, and degradation during growth and development (Park and Seo, 2015). They originally were described in relation to heat shock (Ritossa, 1962) but are induced also by various stresses, such as cold, osmotic stress, anoxia, salinity, water stress, UV-B light etc. (Park and Seo, 2015). Several proposed models tried to explain the increased expression of HSPs in response to heat stress. The protein unfolding model suggests that heat-damaged proteins in the cytoplasm presumably recruit the cytoplasmic chaperones, thereby allowing inactive heat shock transcription factors (HSFs) to undergo phosphorylation, oligomerization, and translocation to the nucleus to transcribe HSP genes (Morimoto, 1998). The plasma membrane model suggests that heat-induced increase of membrane fluidity and changes in microdomain organization (i.e., lipid rafts) can generate a significant HSP expression (Horváth et al., 1998; Vigh et al., 2007; Saidi et al., 2009). Saidi et al. (2009) showed that increased expression of HSPs is strongly dependent on a preceding Ca^2+^ transient through Ca^2+^ permeable channel, which is also activated by membrane fluidizers. Changes in membrane fluidity have been previously found to impact ion channel activity (Collins et al., 1993). This may explain the increased HSP expression after diethyl ether treatment in our study, because diethyl ether also increased [Ca^2+^]_cyt_ (Figure 6) and ion leakage at room temperature (Figure 10), membrane fluidity, and disrupted lipid rafts (Lerner, 1997; Pavel et al., 2020). Significantly upregulated GO-MF categories of “calcium ion binding” and “calmodulin binding” are in accordance with the role of Ca^2+^ in signaling during anesthesia (Figure 2F). This finding is partially interesting in the view of recent studies showing that diethyl ether blocked wound-induced glutamate-dependent Ca^2+^ transient and systemic response mediated by GLR channels in *A. thaliana* (Jakšová et al., 2021) and *D. muscipula* (Scherzer et al., 2022). Different kinetics of these [Ca^2+^]_cyt_ responses suggest participation of two different Ca^2+^ channels. This different effect of diethyl ether on Ca^2+^ transient in response to wounding in etherized plants and ether itself is another piece of evidence of complicated network of so called calcium signature (McAinsh and Pittman, 2009). A third model for HSPs induction suggests the role of reactive oxygen species (ROS). In addition to their detrimental character, ROS are also considered as important signaling molecules, which can induce expression of HSPs (Volkov et al., 2006; Scarpeci et al., 2008; Driedonks et al., 2015). ROS formation after plant exposure to diethyl ether anesthesia was recently documented in *A. thaliana* roots (Yokawa et al., 2018, 2019).

The role of HSPs in multiple stress responses might explain the phenomenon of cross-tolerance or priming, where exposure to a certain stress factors improves tolerance to a subsequent different stress factors in plants (Bowler and Fluhr, 2000; Driedonks et al., 2015; Nair et al., 2022). In animals, such anesthetic preconditioning has been also suggested (Sergeev et al., 2004; Kitahata et al., 2008; Pagel, 2008). Therefore, we investigated whether GVA application and HSP induction can protect plant against subsequent heat stress by measuring a fast chlorophyll *a* fluorescence induction. One of the target site for elevated temperature-induced damage is PSII. Heat stress induces detachment of OEC proteins, loss of cofactors (Mn) from PSII and cleavage of D1 protein (Yoshioka et al., 2006). Our results showed increased thermal stability of PSII in plants exposed to diethyl ether for 5.5 h prior to subsequent heat–stress (Figure 8), indicating that diethyl ether provided protective role against heat–stress probably through induced HSPs. Indeed, the experiments of other authors demonstrated that HSPs can associate with thylakoids and protects O_2_ evolution and OEC proteins of PSII against heat stress. It has been considered that HSPs in chloroplasts do not participate in the repair of stress-related damage but rather function as molecular chaperons to prevent protein denaturation and aggregation (Downs et al., 1999; Allakhverdiev et al., 2008). Saidi et al. (2009) also documented increased thermotolerance of PSII after priming with membrane fluidizer benzylalcohol in *Physcomitrella patens,* what is in accordance with our study.

Other class of genes/proteins upregulated by diethyl ether is involved in vesicle-mediated transport (Figure 2D). This finding is interesting and in accordance with a previous study, where it has been shown that the 15% diethyl ether and 1% lidocaine treatments slowed the rate of endocytic vesicle recycling in *Arabidopsis* root epidermal cells (Yokawa et al., 2018). Although the mechanism involved remains unclear, these results indicate that anesthetics alter normal membrane properties and interact with vesicle trafficking in plants (Yokawa et al., 2019). The important implication of this finding in plants is applicable for presynaptic release of neurotransmitter in animal neurons. Worms with altered sensitivity to GVA were found to have mutation in syntaxin forming the SNARE complex that regulates presynaptic neurotransmitter release (van Swinderen et al., 1999). Interestingly, genes encoding “SNARE binding” were among enriched GO-MF categories also in *Arabidopsis* (Figure 2F).

Our recent study has found inhibition of chlorophyll accumulation during de-etiolization under diethyl ether anesthesia in garden cress (Yokawa et al., 2018). In our experiments with circadian grown plants, the chlorophyll *a* + *b* concentration was not significantly different (data not shown) due to the high amount of chlorophylls which were pre-synthetized 6–7 weeks before 5.5 h diethyl ether treatment. However, our analysis found that chlorophyll biosynthesis is transcriptionally downregulated also in mature circadian-grown plants, because 26 out of 34 genes encoding proteins involved in tetrapyrrole biosynthesis were significantly downregulated (Figure 4). This was mirrored also on protein level (Figure 4). Concomitantly, the transcription of many photosynthesis-related genes was also downregulated (Figure 4). If this was a result of plastid to nucleus retrograde redox signaling (e.g., through changes of redox state of plastoquinone pool indicating by differences in 1-qP, Figure 7) or direct effect of diethyl ether remains unknown. Recently, it was found that increased [Ca^2+^]_cyt_, which was also documented in this study (Figure 6), is responsible for repression of *LHCB* genes mediated by MAP kinases phosphorylation of ABI4 (Guo et al., 2016). Chlorophyll metabolism is tightly regulated by different factors like heat, light, cold, phytohormones and is also non-specific indicator of any plant stress (Kruse et al., 1997; Tewari and Tripathy, 1998; Matsumoto et al., 2004; Mohanty et al., 2006; Yaronskaya et al., 2006; Kobayashi and Masuda, 2016). It is known for decades that GVA and lidocaine inhibited photosynthetic reactions in isolated chloroplasts (Wu and Berkowitz, 1991; Nakao et al., 1998). In our study, we measured photosynthetic reactions by *in vivo* chlorophyll *a* fluorescence in intact plants and we did not find anesthesia-induced inhibition of photosynthesis. On the contrary, after reaching steady-state conditions, the rate of photosynthetic electron transport (expressed as Ф_PSII_) was even slightly higher in etherized plants (Figure 7).

**Figure 11:**
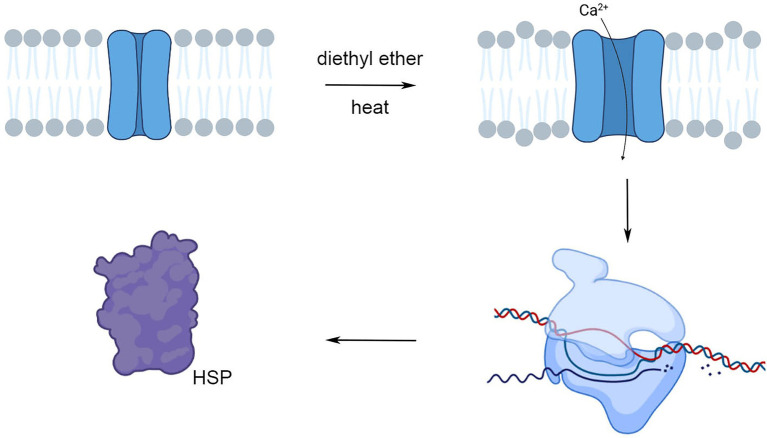
The hypothesis about similar action of diethyl ether anesthesia and heat stress on HSP expression. Both, anesthesia and heat stress fluidizes a membrane and affect Ca^2+^ channel function. The consequent Ca^2+^ entry into the cytoplasm triggers signaling cascade leading to the changes of gene expression (e.g., induction of HSPs). Created in BioRender.com.

Our study showed that plants under anesthesia with diethyl ether had not only decreased ability to sense their environment (Yokawa et al., 2018; Pavlovič et al., 2020; Jakšová et al., 2021; Scherzer et al., 2022) but surprisingly also strongly reprogrammed gene expression. The possible underlying mechanism involved Ca^2+^ entry into the cells through the effect on plasma membranes and thus resembles the effect of heat stress (Figure 11). While the effect on primary photosynthetic reactions is rather marginal in etherized plants, exposure of plants to anesthetic may protect the PSII against subsequent heat stress through the effect of cross-tolerance or priming. This study has shown that the effects of anesthesia and term anesthesia go far beyond consciousness and modern medicine having wider implications for a variety of organisms and deserve our further attention.

## Data availability statement

The datasets presented in this study can be found in online repositories. The names of the repository/repositories and accession number(s) can be found at: https://datadryad.org/stash, doi: 10.5061/dryad.wm37pvmqq.

## Author contributions

AP designed the research, analyzed the data, and wrote the manuscript. AP and AM provided material and financial support. JJ did Western blots and isolated RNA for RNA-seq experiments. PR performed proteomic analysis. MŠ and ZK measured chlorophyll *a* fluorescence and relative conductivity. MR and AP measured [Ca^2+^]_cyt_ signals. All authors contributed to the article and approved the submitted version.

## Funding

The study was supported by the Czech Science Foundation Agency GAČR (21-03593S). CIISB, Instruct-CZ Center of Instruct-ERIC EU consortium, funded by MEYS CR infrastructure project LM2018127, is also gratefully acknowledged for the financial support of the measurements at the CF Prot.

## Conflict of interest

The authors declare that the research was conducted in the absence of any commercial or financial relationships that could be construed as a potential conflict of interest.

## Publisher’s note

All claims expressed in this article are solely those of the authors and do not necessarily represent those of their affiliated organizations, or those of the publisher, the editors and the reviewers. Any product that may be evaluated in this article, or claim that may be made by its manufacturer, is not guaranteed or endorsed by the publisher.

## Supplementary material

SUPPLEMENTARY TABLE S1 The complete list of significantly upregulated and downregulated differentially expressed genes (DEGs) in *A. thaliana* arranged from the highest to lowest log_2_ fold change (*p* < 0.05).

SUPPLEMENTARY TABLE S2 The complete list of significantly enriched GO terms at *p* < 0.05 arranged from the lowest to highest adjusted *p* values (*p*_adj_) in *A. thaliana*.

SUPPLEMENTARY TABLE S3 The complete list of significantly upregulated and downregulated differentially expressed proteins (DEPs) in *A. thaliana* arranged from the highest to lowest log_2_ fold change (*p* < 0.05).

SUPPLEMENTARY VIDEO S1 Luminescence in whole *A. thaliana* rosette expressing the APOAEQUORIN gene with coelenterazine in response to diethyl ether application at time point 1 min. The plants were sprayed with coelenterazine and the signal is indicator of [Ca^2+^]_cyt_. Photons were captured in photon-counting mode with a 5 min acquisition time.

SUPPLEMENTARY VIDEO S2 Luminescence in whole *A. thaliana* rosette expressing the APOAEQUORIN gene without coelenterazine in response to diethyl ether application at time point 1 min. The plants were not sprayed with coelenterazine before measurements and the possible signal is indicator of ROS production. Photons were captured in photon-counting mode with a 5 min acquisition time.

## References

[ref1] Allakhverdiev S. I. Kreslavski V. D. Klimov V. V. Los D. A. Carpentier R. Mohanty P. (2008). Heat stress: an overview of molecular responses in photosynthesis. Photosynth. Res. 98, 541–550. doi: 10.1007/s11120-008-9331-0, PMID: 18649006

[ref2] Böhm J. Scherzer S. (2021). Signaling and transport processes related to the carnivorous lifestyle of plants living on nutrient-poor soil. Plant Physiol. 187, 2017–2031. doi: 10.1093/plphys/kiab297, PMID: 35235668 PMC8890503

[ref3] Bowler C. Fluhr R. (2000). The role of calcium and activated oxygens as signals for controlling cross-tolerance. Trends Plant Sci. 5, 241–246. doi: 10.1016/s1360-1385(00)01628-9, PMID: 10838614

[ref4] Cheng Z. Luan Y. Meng J. Sun J. Tao J. Zhao D. (2021). WRKY transcription factor response to high-temperature stress. Plants 10:2211. doi: 10.3390/plants10102211, PMID: 34686020 PMC8541500

[ref5] Coghlan M. Richards E. Shaik S. Rossi P. Vanama R. B. Ahmadi S.. (2018). Inhalation anesthetics induce neuronal protein aggregation and use affect ER trafficking. Sci. Rep. 8:5275. doi: 10.1038/s41598-018-23335-0, PMID: 29588456 PMC5869676

[ref6] Collins A. C. Wehner J. M. Wilson W. R. (1993). Animal models for alcoholism: genetic strategies and neurochemical mechanisms. Biochem. Soc. Symp. 59, 173–191. PMID: 8192685

[ref7] Cox J. Mann M. (2008). MaxQuant enables high peptide identification rates, individualized p.p.b.-range mass accuracies and proteome-wide protein quantification. Nat. Biotechnol. 26, 1367–1372. doi: 10.1038/nbt.1511, PMID: 19029910

[ref8] Cox J. Neuhauser N. Michalski A. Scheltema R. A. Olsen J. V. Mann M. (2011). Andromeda: a peptide search engine integrated into the MaxQuant environment. J. Proteome Res. 10, 1794–1805. doi: 10.1021/pr101065j, PMID: 21254760

[ref9] De Luccia T. P. B. (2012). *Mimosa pudica*, *Dionaea muscipula* and anesthetics. Plant Signal. Behav. 7, 1163–1167. doi: 10.4161/psb.21000, PMID: 22899087 PMC3489652

[ref10] Demidchik V. Straltsova D. Medvedev S. S. Pozhvanov G. A. Sokolik A. Yurin V. (2014). Stress-induced electrolyte leakage: the role of K+-permeable channels and involvement in programmed cell death and metabolic adjustment. J. Exp. Bot. 65, 1259–1270. doi: 10.1093/jxb/eru004, PMID: 24520019

[ref11] Downs C. A. Coleman J. S. Heckathorn S. A. (1999). The chloroplast 22-ku heat-shock protein: a lumenal protein that associates with the oxygen evolving complex and protects photosystem II during heat stress. J. Plant Physiol. 155, 477–487. doi: 10.1016/S0176-1617(99)80042-X

[ref12] Driedonks N. Xu J. Peters J. L. Park S. Rieu I. (2015). Multi-level interactions between heat shock factors, heat shock proteins, and the redox system regulate acclimation to heat. Front. Plant Sci. 6:999. doi: 10.3389/fpls.2015.00999, PMID: 26635827 PMC4647109

[ref13] Franks N. P. (2006). Molecular targets underlying general anesthesia. Br. J. Pharmacol. 147, S72–S81. doi: 10.1038/sj.bjp.0706441, PMID: 16402123 PMC1760740

[ref14] Franks N. P. Lieb W. R. (1984). Do general anesthetics act by competitive binding to specific receptors? Nature 310, 599–601. doi: 10.1038/310599a0, PMID: 6462249

[ref15] Guissé B. Srivastava A. Strasser R. J. (1995). The polyphasic rise of the chlorophyll a fluorescence (O-K-J-I-P) in heat stressed leaves. Arch. Sci. Genev. 48, 147–160.

[ref01] Guo H. Feng P. Chi W. Sun X. Xu X. Li Y.. (2016). Plastid-nucleus communication involves calcium-modulated MAPK signalling. Nature Comm. 7:12173. doi: 10.1038/ncomms12173 PMC4942575 27399341

[ref16] Horváth I. Glatz A. Varvasovszki V. Török Z. Páli T. Balogh G.. (1998). Membrane physical state controls the signaling mechanism of the heat shock response in *Synechocystis* PCC 6803: identification of *hsp17* as a “fluidity gene”. Proc. Natl. Acad. Sci. U. S. A. 95, 3513–3518. doi: 10.1073/pnas.95.7.3513, PMID: 9520397 PMC19867

[ref17] Ilík P. Špundová M. Šicner M. Melkovičová H. Kučerová Z. Krchňák P.. (2018). Estimating heat tolerance of plants by ion leakage: a new method based on gradual heating. New Phytol. 218, 1278–1287. doi: 10.1111/nph.15097, PMID: 29573424

[ref18] Jakšová J. Rác M. Bokor B. Petřík I. Novák O. Reichelt M.. (2021). Anesthetic diethyl ether impairs systemic electrical and jasmonate signaling in *Arabidopsis thaliana*. Plant Physiol. Biochem. 169, 311–321. doi: 10.1016/j.plaphy.2021.11.019, PMID: 34826706

[ref19] Kelz M. B. Mashour G. A. (2019). The biology of general anesthesia from paramecium to primate. Curr. Biol. 29, R1199–R1210. doi: 10.1016/j.cub.2019.09.071, PMID: 31743680 PMC6902878

[ref20] Kiep V. Vadassery J. Lattke J. Maaß J.-P. Boland W. Peiter E.. (2015). Systemic cytosolic Ca^2+^ elevation is activated upon wounding and herbivory in Arabidopsis. New Phytol. 207, 996–1004. doi: 10.1111/nph.13493 25996806

[ref21] Kitahata H. Nozaki J. Kawahito S. Tomino T. Oshita S. (2008). Low-dose sevoflurane inhalation enhances late cardioprotection from anti-ulcer drug geranygeranylacetone. Anesth. Analg. 107, 755–761. doi: 10.1213/ane.0b013e31817f0e61, PMID: 18713879

[ref22] Kobayashi K. Masuda T. (2016). Transcriptional regulation of tetrapyrrole biosynthesis in *Arabidopsis thaliana*. Front. Plant Sci. 7:1811. doi: 10.3389/fpls.2016.01811, PMID: 27990150 PMC5130987

[ref23] Kruse E. Grimm B. Beator J. Kloppstech K. (1997). Developmental and circadian control of the capacity for δ-aminolevulinic acid synthesis in green barley. Planta 202, 235–241. doi: 10.1007/s004250050124

[ref24] Lazár D. Pospíšil P. Nauš J. (1997). Decrease of fluorescence intensity after the K step in chlorophyll a fluorescence induction is suppressed by electron acceptors and donors to photosystem 2. Photosynthetica 37, 255–265. doi: 10.1023/A:1007112222952

[ref25] Lerner R. A. (1997). A hypothesis about the endogenous analogue of general anesthesia. Proc. Natl. Acad. Sci. U. S. A. 94, 13375–13377. doi: 10.1073/pnas.94.25.13375, PMID: 9391028 PMC33784

[ref26] Matsumoto F. Obayashi T. Sasaki-Sekimoto Y. Ohta H. Takamiya K. Masuda T. (2004). Gene expression profiling of the tetrapyrrole metabolic pathway in Arabidopsis with a mini-array system. Plant Physiol. 135, 2379–2391. doi: 10.1104/pp.104.042408, PMID: 15326282 PMC520805

[ref27] Maxwell K. Johnson G. N. (2000). Chlorophyll fluorescence—a practical guide. J. Exp. Bot. 51, 659–668. doi: 10.1093/jxb/51.345.659, PMID: 10938857

[ref28] McAinsh M. R. Pittman J. K. (2009). Shaping the calcium signature. New Phytol. 181, 275–294. doi: 10.1111/j.1469-8137.2008.02682.x 19121028

[ref29] Meyer H. (1899). Zur Theorie der Alkoholnarkose. Arch. Exp. Pathol. Pharmakol. 42, 109–118. doi: 10.1007/BF01834479

[ref30] Milne A. Beamish T. (1999). Inhalational and local anesthetics reduce tactile and thermal responses in *Mimosa pudica*. Can. J. Anaesth. 46, 287–289. doi: 10.1007/BF03012612, PMID: 10210057

[ref31] Mohanty S. Grimm B. Tripathy B. C. (2006). Light and dark modulation of chlorophyll biosynthetic genes in response to temperature. Planta 224, 692–699. doi: 10.1007/s00425-006-0248-6, PMID: 16523349

[ref32] Morimoto R. I. (1998). Regulation of the heat shock transcriptional response: cross talk between a family of heat shock factors, molecular chaperones, and negative regulators. Genes Dev. 12, 3788–3796. doi: 10.1101/gad.12.24.3788, PMID: 9869631

[ref33] Mousavi S. A. R. Chauvin A. Pascaud F. Kellenberger S. Farmer E. E. (2013). Glutamate receptor-like genes mediate leaf-to-leaf wound signals. Nature 500, 422–426. doi: 10.1038/nature12478, PMID: 23969459

[ref34] Nair A. Bhukya D. P. N. Sunkar R. Chavali S. Allu A. D. (2022). Molecular basis of priming-induced acquired tolerance to multiple abiotic stresses in plants. J. Exp. Bot. 73, 3355–3371. doi: 10.1093/jxb/erac089, PMID: 35274680

[ref35] Nakao H. Ogli K. Yokono S. Ono J. Miyatake A. (1998). The effect of volatile anesthetics on light-induced phosphorylation in spinach chloroplasts. Toxicol. Lett. 100-101, 135–138. doi: 10.1016/s0378-4274(98)00177-5, PMID: 10049133

[ref36] Overton C. E. (1901). Studien über die Narkose Zugleich ein Beitrag zur Allgemeinen Pharmakologie. Jena, Germany: Fischer Verlag.

[ref37] Pagel P. S. (2008). Induction of heat shock protein 70 and preconditioning by sevoflurane: a potent protective interaction against myocardial ischemia-reperfusion injury. Anesth. Analg. 107, 742–745. doi: 10.1213/ane.0b013e31817f6d40, PMID: 18713875

[ref38] Park C.-J. Seo Y.-S. (2015). Heat shock proteins: a review of the molecular chaperones for plant immunity. Plant Pathol. J. 31, 323–333. doi: 10.5423/PPJ.RW.08.2015.0150, PMID: 26676169 PMC4677741

[ref39] Pavel M. A. Petersen N. Wang H. Lerner R. A. Hansen S. B. (2020). Studies on the mechanism of general anesthesia. Proc. Natl. Acad. Sci. U. S. A. 117, 13757–13766. doi: 10.1073/pnas.2004259117, PMID: 32467161 PMC7306821

[ref40] Pavlovič A. Libiaková M. Bokor B. Jakšová J. Petřík I. Novák O.. (2020). Anaesthesia with diethyl ether impairs jasmonate signaling in the carnivorous plant Venus flytrap (*Dionaea muscipula*). Ann. Bot. 125, 173–183. doi: 10.1093/aob/mcz177, PMID: 31677265 PMC6948209

[ref41] Raudvere U. Kolberg L. Kuzmin I. Arak T. Adler P. Peterson H.. (2019). G:profiler: a web server for functional enrichment analysis and conversions of gene lists (2019 update). Nucleic Acids Res. 47, W191–W198. doi: 10.1093/nar/gkz369, PMID: 31066453 PMC6602461

[ref42] Ritossa F. (1962). A new puffing pattern induced by temperature shock and DNP in drosophila. Experientia 18, 571–573. doi: 10.1007/BF02172188

[ref43] Saidi Y. Finka A. Muriset M. Bromberg Z. Weiss Y. G. Maathuis F. J. M.. (2009). The heat shock response in moss plants is regulated by specific calcium-permeable channels in the plasma membrane. Plant Cell 21, 2829–2843. doi: 10.1105/tpc.108.065318, PMID: 19773386 PMC2768932

[ref44] Scarpeci T. E. Zanor M. I. Valle E. M. (2008). Investigating the role of plant heat shock proteins during oxidative stress. Plant Signal. Behav. 3, 856–857. doi: 10.4161/psb.3.10.6021, PMID: 19704521 PMC2634396

[ref45] Schägger H. (2006). Tricine–SDS-PAGE. Nat. Protoc. 1, 16–22. doi: 10.1038/nprot.2006.4 17406207

[ref46] Scherzer S. Huang S. Iosip A. Kreuzer I. Yokawa K. Al-Rasheid K. A. S.. (2022). Ether anesthetics prevents touch-induced trigger hair calcium-electrical signals excite the Venus flytrap. Sci. Rep. 12:2851. doi: 10.1038/s41598-022-06915-z, PMID: 35181728 PMC8857258

[ref47] Sergeev P. da Silva R. Lucchinetti E. Zaugg K. Pasch T. Schaub M. C.. (2004). Trigger-dependent gene expression profiles in cardiac preconditioning. Evidence for distinct genetic programs in ischemic and anesthetic preconditioning. Anesthesiology 100, 474–488. doi: 10.1097/00000542-200403000-00005, PMID: 15108959

[ref48] Srivastava A. Guissé B. Greppin H. Strasser R. J. (1997). Regulation of antenna structure and electron transport in photosystem II of Pisum sativum under elevated temperature probed by the fast polyphasic chlorophyll *a* fluorescence transient: OKJIP. Biochim. Biophys. Acta-Bioenerget. 1320, 95–106. doi: 10.1016/S0005-2728(97)00017-0

[ref49] Tang P. Xu Y. (2002). Large-scale molecular dynamics simulations of general anesthetic effects on the ion channel in the fully hydrated membrane: the implication of molecular mechanisms of general anesthesia. Proc. Natl. Acad. Sci. U. S. A. 99, 16035–16040. doi: 10.1073/pnas.252522299, PMID: 12438684 PMC138560

[ref02] Tewari A. K. Tripathy B. C. (1998). Temperature-stress-induced impairment of chlorophyll biosynthetic reactions in cucumber and wheat. Plant Physiol. 117, 851–858. doi: 10.1104/pp.117.3.851 9662527 PMC34939

[ref50] Toyota M. Spencer D. Sawai-Toyota S. Jiaqi W. Zhang T. Koo A. J.. (2018). Glutamate triggers long-distance, calcium-based plant defense signaling. Science 361, 1112–1115. doi: 10.1126/science.aat7744, PMID: 30213912

[ref51] Upton D. H. Popovic K. Fulton R. Kassiou M. (2020). Anesthetic-dependent changes in gene expression following acute and chronic exposure in the rodent brain. Sci. Rep. 10:9366. doi: 10.1038/s41598-020-66122-6, PMID: 32518252 PMC7283325

[ref52] Urban B. W. Bleckwenn M. (2002). Concepts and correlations relevant to general anaesthesia. Br. J. Anaesth. 89, 3–16. doi: 10.1093/bja/aef164, PMID: 12173238

[ref53] van Swinderen B. Saifee O. Shebester L. Roberson R. Nonet M. L. Crowder C. M. (1999). A neomorphic syntaxin mutation blocks volatile-anesthetic action in *Caenorhabditis elegans*. Proc. Natl. Acad. Sci. U. S. A. 96, 2479–2484. doi: 10.1073/pnas.96.5.2479, PMID: 10051668 PMC26810

[ref54] Vigh L. Horváth I. Maresca B. Harwood J. L. (2007). Can the stress protein response be controlled by ‘membrane-lipid therapy’? Trends Biochem. Sci. 32, 357–363. doi: 10.1016/j.tibs.2007.06.009, PMID: 17629486

[ref55] Volkov R. A. Panchuk I. I. Mullineaux P. M. Schöffl F. (2006). Heat stress-induced H_(2)_O_(2)_ is required for effective expression of heat shock genes in Arabidopsis. Plant Mol. Biol. 61, 733–746. doi: 10.1007/s11103-006-0045-4, PMID: 16897488

[ref56] Wiśniewski J. R. Zougman A. Nagaraj N. Mann M. (2009). Universal sample preparation method for proteome analysis. Nat. Methods 6, 359–362. doi: 10.1038/nmeth.1322 19377485

[ref57] Wu W. Berkowitz G. A. (1991). Lidocaine and ATPase inhibitor interaction with the chloroplast envelope. Plant Physiol. 97, 1551–1557. doi: 10.1104/pp.97.4.1551, PMID: 16668583 PMC1081199

[ref58] Yaronskaya E. Vershilovskaya I. Poers Y. Alawady A. E. Averina N. Grimm B. (2006). Cytokinin effects on tetrapyrrole biosynthesis and photosynthetic activity in barley seedlings. Planta 224, 700–709. doi: 10.1007/s00425-006-0249-5, PMID: 16506064

[ref59] Yokawa K. Kagenishi T. Baluška F. (2019). Anesthetics, anesthesia, and plants. Trends Plant Sci. 24, 12–14. doi: 10.1016/j.tplants.2018.10.006, PMID: 30446303

[ref60] Yokawa K. Kagenishi T. Pavlovič A. Gall S. Weiland M. Mancuso S.. (2018). Anesthetics stop diverse plant organ movements, affect endocytic vesicle recycling and ROS homeostasis, and block action potentials in Venus flytraps. Ann. Bot. 122, 747–756. doi: 10.1093/aob/mcx155, PMID: 29236942 PMC6215046

[ref61] Yoshioka M. Uchida S. Mori H. Komayama K. Ohira S. Morita N.. (2006). Quality control of photosystem II. Cleavage of reaction center D1 protein in spinach thylakoids by Fts H protease under moderate heat stress. J. Biol. Chem. 281, 21660–21669. doi: 10.1074/jbc.M602896200 16735503

